# Metastasis directed radiotherapy versus standard of care for PSMA-PET diagnosed oligometastatic/oligoprogressive castration resistant prostate cancer

**DOI:** 10.1038/s41598-025-09622-7

**Published:** 2025-07-25

**Authors:** Laura Bauersachs, Marcus Beck, Christian Furth, Felix Mehrhof, Holger Amthauer, Pirus Ghadjar, Julian M. M. Rogasch, Maria de Santis, Rana Tahbaz, Daniel Zips, Sebastian Zschaeck

**Affiliations:** 1 Department of Radiation Oncology, Charité – Universitätsmedizin Berlin, Corporate Member of Freie Universität Berlin, Humboldt-Universität zu Berlin, and Berlin Institute of Health, Augustenburger Platz 1, 13353 Berlin, Germany; 2 Department of Nuclear Medicine, Charité – Universitätsmedizin Berlin, Corporate Member of Freie Universität Berlin, Humboldt-Universität zu Berlin, and Berlin Institute of Health, Berlin, Germany; 3 Department of Urology, Charité – Universitätsmedizin Berlin, Corporate Member of Freie Universität Berlin, Humboldt-Universität zu Berlin, and Berlin Institute of Health, Berlin, Germany; 4 Charité - Universitätsmedizin Berlin, German Cancer Consortium (DKTK), Partner Site Berlin, and German Cancer Research Center (DKFZ), 69120 Berlin, Heidelberg, Germany

**Keywords:** PSMA PET, Positron emission tomography, Castration resistant prostate cancer, Oligometastatic prostate cancer, Oligoprogressive prostate cancer, Ablative radiotherapy, Stereotactic irradiation, Metastasis directed radiotherapy, Second line androgen deprivation therapy, Cancer imaging, Prostate cancer

## Abstract

In recent years there has been a growing interest in metastasis directed radiotherapy (MDRT) in hormone-sensitive oligometastatic prostate cancer. The role of MDRT in castration resistant prostate cancer (CRPC) patients remains controversial. Our study retrospectively compared MDRT to standard of care (SOC) in oligometastatic/oligoprogressive CRPC patients staged by PSMA PET CT. Patients either received SOC or MDRT with continuation of androgen deprivation therapy (ADT). Investigated endpoints contained biochemical progression-free survival (bPFS), overall survival (OS) and freedom from second line therapy (FFSLT). In this retrospective monocenter study, all patients with PSMA PET between January 2014 and July 2018 were screened. 55 oligometastatic/oligoprogressive CRPC patients were identified and further analysed, 34 received MDRT and 21 SOC. Baseline characteristics were similarly distributed between groups. Kaplan–Meier estimates suggested a trend for improved bPFS (*p* = 0.10) and improved OS (*p* = 0.01) by MDRT. Additionally, FFSLT was significantly prolonged in MDRT patients (*p* = 0.006). Multivariate cox regression analyses revealed MDRT as the only parameter that was significantly associated with bPFS (hazard ratio 0.36, *p* = 0.048) and OS (hazard ratio 0.14, *p* = 0.006). No high-grade radiation induced toxicities were observed. In our study MDRT was a well-tolerated treatment option with low toxicity in oligometastatic/oligoprogressive CRPC patients, resulting in improved OS and freedom from second-line therapy and a potential improvement of bPFS compared to SOC.

## Introduction

Since the introduction of 68-Ga-Prostate-specific-membran-Antigen-positron- Computertomography (PSMA-PET) it is possible to identify prostate cancer patients in an early oligometastatic state of their disease^1^. PSMA-PET showed superiority to conventional imaging modalities and other previously used PET tracers regarding sensitivity and specifity for lymphnode and distant metastasis detection^2,3^. In addition, there has been a change of treatment paradigm in metastatic prostate cancer. According to the hypothesis of the oligometastatic state of disease, first described by Hellman and Weichselbaum, the oligometastatic state can be considered as an intermediate stage between a localised disease and a disease which has progressed to a systemic state^4^. Most studies define oligometastatic prostate cancer (OMPC) as a status of disease with 1–5 lymph node or bone metastases^5,6^.

In recent years there has been a growing interest in metastasis directed radiotherapy (MDRT) such as stereotactic ablative radiotherapy (SABR) in oligometastatic prostate cancer.

Several prospective randomized phase II-trials have proved that patients with oligometastatic prostate cancer had a benefit from SABR to their metastatic lesions regarding progression-free- survival (PFS) compared to surveillance alone. In hormone sensitive disease an additional aim can be to delay the start of androgen deprivation therapy (ADT)^7,8^. In most trails only hormone sensitive prostate cancer patients were enrolled.

It has been shown that patients with castration resistant disease experience a disease progression while undergoing ADT due to androgen receptor alterations^9^. Historically, this state of disease is associated with a poor overall survival below two years^10^. The standard of care for castration resistant prostate cancer is the intensification of systemic therapy such as ADT, especially Androgen Receptor Pathway Inhibitor (ARPI), chemotherapy, immunotherapy, Lutetium-177-labeled prostate-specific membrane antigen-targeted radioligand therapy^11^, olaparib and other PARP inhibitors^12^ or Radium-223 dichloride in patients with bone metastases^13^. These means of therapeutic intensification showed an improvement of overall survival (OS). For example, The COU-AA-301 trial was able to show an OS improvement by arbiraterone compared to placebo with a Median OS of 15.8 versus 11.2 months^14^.

To our knowledge, there is only a limited number of studies on MDRT in oligometastatic or oligoprogressive CRPC patients^15,16^.

With our study we would like to improve our understanding, if CRPC patients staged oligometastatic/oligoprogressive by PSMA-PET CT benefit from MDRT. For comparison we screened all CRPC patients that underwent PSMA-PET and identified patients that had only a limited number of metastases in PET staging. According to the choice of the treating physician, patients were either treated with standard of care or additional MDRT.

Our aim was to analyse and compare these therapeutic approaches regarding important oncological outcome parameters like progression free and overall survival but also parameters with potential importance to quality of life like freedom from second line androgen deprivation therapy (SLADT).

## Results

A total of 55 castration resistant prostate cancer patients with oligometastases or oligoprogressive disease detected by PSMA-PET/CT were included in the analysis. Since most of these patients did not undergo initial PSMA-PET/CT staging, it is not possible to differentiate initial oligometastatic from oligoprogressive disease in this cohort. The patients were grouped by the chosen treatment approach: 34 patients received MDRT (MDRT group). In the control group 21 patients received standard of care. The median follow-up time was 58.7 months.

Patient characteristics are summarized in Table 1. A total of 81 lesions in the MDRT group were detected by PSMA-PET CT compared to 53 lesions in the control group. All patients with nodal disease (N +) had at least one additional non-regional tumor lesion. Bone metastases were more frequently observed in the MDRT group (30 lesions in 16 patients) in comparison to the control group (7 lesions in 6 patients). After baseline PSMA-PET, all patients were treated according to individual choice of the referring physicians and patients´preference.

**Table 1:** Patient characteristics before baseline PSMA-PET/CT.

	Radiotherapy group MW + (Range)	Control group MW + (Range)
Initial tumor presentation		
Initial PSA-Value (ng/ml)	47.5 (2.7–508)	73.52 (2.0–443.0)
Initial Gleason (prostate biopsy)	8.03 (6–9)	8.37 (7–10)
Risk group		
Low risk Intermediate risk High risk	n = 1 (2.94%) n = 3 (8.82%) n = 20 (58.82%)	n = 1 (5.26%) n = 3 (15.79%) n = 8 (42.11%)
N + M +	n = 8 (23.53%) n = 2 (5.88%)	n = 4 (21.05%) n = 3 (15.79%)
UICC stage I UICC stage II	n = 6 (17.65%) n = 3 (8.82%)	n = 2 (10.53%) n = 3 (15.79%)
UICC stage III UICC stage IV	n = 14 (41.18%) n = 11 (32.35%)	n = 6 (31.58%) n = 8 (42.11%)
Charlson Morbidity Index (CCI) 0 Point	n = 27 (79.41%)	n = 15 (71.43%)
CCI 1 point CCI 2 points	n = 5 (14.71%) n = 1 (4.76%)	n = 5 (23.81) n = 0
Primary therapy		
RPE	n = 24 (64.71%)	n = 13 (61.90%)
Primary radiotherapy	n = 6 (17.64%)	n = 2 (9.52%)
Salvage radiotherapy	n = 18 (52.94%)	n = 8 (38.10%)
Primary ADT only	n = 4 (11.76%)	n = 6 (28.57%)
other (RFA/ HIFU etc.)	n = 1 (2.94%)	
Therapy before PSMA-PET/CT		
Bicalutamid/LHRH	n = 34 (100%)	n = 21 (100%)
ARPI before PSMA-PET/CT	n = 1 (2.94%)	n = 0
Cytostatic therapy before PSMA-PET/CT	n = 1 (2.94%)	n = 0
Additional subsequent local therapy before PSMA-PET/CT not confined to prostate (e.g. radiotherapy and/or surgery for bone metastatses)	n = 6 (17.64%)	n = 2 (9.52%)
Patient characteristics before PSMA-PET		
Age at PSMA-PET (years)	71.05 (50–84)	73.28 (65–85)
Time from initial diagnosis to PSMA-PET (months)	82.29 (14–197)	100.14 (9–257)
PSA level at the time of PSMA-PET	7.10 (0.38–100) ng/ml	7.85 (0.04–46.0) ng/ml
PSA doubling time at the time of PSMA-PET	5.29 (0.87–23.7) months	5.52 (1.95–19.6) months
Duration of ADT intake until PSMA-PET	60.32 (6–197) months	56.09 (6–149) months
PSMA-PET findings		
Untreated/relapsed primary tumor	n = 7	n = 11
Pelvic lymphnodes (N)	n = 25 (10 patients)	n = 17 (9 patients)
Extrapelvic lymphnodes (M1a)	n = 19 (12 patients)	n = 18 (7 patients)
Bone metastases (M1b)	n = 30 (16 patients)	n = 7 (6 patients)
Number of PSMA avid lesions per patient	2.4	2.5

Detailed treatment characteristics are shown in Table 2.

**Table 2:** Treatment characteristics after baseline PSMA-PET/CT.

	MDRT group	Control group
PSA level 6 months after PSMA-PET	3.25 ng/ml (range: 0.01–27.07)	7.80 ng/ml (range:0.01–60.0)
Subsequent therapy after PSMA-PET		
Abiraterone	n = 20 (58.82%)	n = 9 (42.86%)
Enzalutamide	n = 14 (41.18%)	n = 8 (38.10%)
Abiraterone and Enzalutamide	n = 9 (26.47%)	n = 4 (19.05%)
Apalutamid	n = 1 (2.94%)	n = 1 (4.76%)
Not recieved ARPI	n = 8 (23.53%)	n = 7 (33.33%)
Zoledron acid/ Denosumab	n = 9 (26.47%)	n = 8 (38.10%)
Docetaxel	n = 12 (35.29%)	n = 8 (38.10%)
Cabazitaxel	n = 8 (23.53%)	n = 5(23.80%)
Palliative RT (a.e. bone metastasis during further follow up)	n = 6 (17.65%)	n = 5 (23.80%)
Radium	n = 6 (17.65%)	n = 0
PSMA-directed Lutetium-177 radio-ligand therapy	n = 2 (5.88%)	n = 5 (23.80%)
Subsequent radiotherapy after second PET	n = 24 (70.59%)	
Symptomatic metastasis in the course	n = 3 (8.8%)	n = 4 (19.05%)

MDRT was performed either as SBRT (n = 16, 47%) or high dose EBRT (n = 13, 38%) or a combination of both (n = 5, 15%) to all metastatic sites during ongoing ADT. There was no grade 2–4 toxicity related to MDRT.

The median bPFS was 33.0 months (range: 8–90 months) in the MDRT group compared to 23.4 months (range: 5–83 months) in the control group. The median OS was 63.3 months (range: 30–111 months) in the MDRT group and 51.2 months (range: 10–97 months) in the control group.

**Fig. 1:**
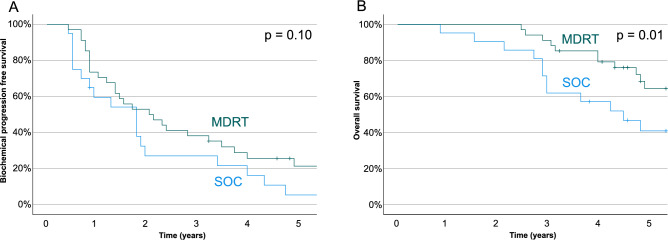
Kaplan Meier estimates for patients with standard of care (SOC) and metastasis directed radiotherapy (MDRT), (**A**) Biochemical progression free survival and (**B**) Overall survival of patients.

Figure 1 shows Kaplan–Meier plots for both endpoints stratified by treatment approach. There was a trend for improved bPFS in the MDRT group (*p* = 0.1) and a significantly improved OS in the MDRT group compared to the control group (*p* = 0.01). In the MDRT group 3-year OS was 93% compared to 63% in the control group.

Regarding biochemical response (BR) after 6 months, a decrease of PSA value of ≥ 50% after baseline PSMA-PET occurred in 20 patients of the MDRT group (59%) vs 7 patients in the control group (37%).

**Fig. 2:**
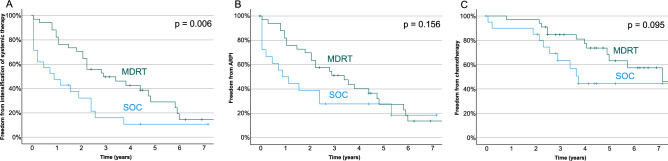
Kaplan Meier estimates for patients with standard of care (SOC) and metastasis directed radiotherapy (MDRT). (**A**) Freedom of intensification of systemic therapy according to treatment group, intensification consisting either of Androgen receptor pathway inhibitors (ARPI), depicted in B or cytostatic chemotherapy, depicted in C.

Freedom from second line therapy (FFSLT) was significantly longer in the MDRT group compared to the control group (*p* = 0.006, Fig. 2 A). FFSLT at 2 years was 73.5% (in the MDT group compared to 28.5% (n = 6 of 21) in the control group. When analyzing specific second line treatment regimes, there was no significant difference regarding FFARPI (*p* = 0.156, Fig. 2B) and a trend for improved FFC (*p* = 0.095, Fig. 2C).

Clinical parameters with known prognostic significance and treatment group were analysed by cox regression analyses. Results of univariate and multivariate analyses are presented in Table 3. There was no association of any parameter with bPFS in univariate cox regression analyses. Upon multivariate testing a significant correlation between bPFS and MDRT (HR = 0.36, 95% CI 0.13–0.94, *p* = 0.048) was observed.

**Table 3:** Univariate and multivariate analysis of prognostic parameters and treatment approach.

Parameter	Univariate cox regression analysis		multivariate cox regression analysis	
	HR	*p*	HR	*p*
Progression free survival				
Radiotherapy	0.62 (0.34–1.12)	0.114	**0.36 (0.13–0.94)**	**0.048**
Age	1.03 (0.98–1.08)	0.229	1.01 (0.93–1.11)	0.73
Time interval initial diagnosis to PSMA-PET	1.00 (0.96–1.01)	0.995	1.00 (0.99–1.02)	0.37
Initial PSA	1.002 (1.00–1.01)	0.088	1.00 (0.99–1.01)	0.06
Gleason score	0.96 (0.67–1.37)	0.825	0.78 (0.39–1.51)	0.46
Initial tumor stage	1.05 (0.74–1.49)	0.791	1.16 (0.67–2.00)	0.587
Charlson comorbidity index	1.06 (0.54–2.05)	0.875	1.28 (0.53–3.09)	0.586
Duration of ADT until PSMA-PET	1.00 (0.99–1.01)	0.744	0.99 (0.98–1.01)	0.220
PSA at PSMA-PET	1.00 (0.95–1.04)	0.864	1.06 (0.93–1.22)	0.387
PSA doubling time	1.02 (0.96–1.07)	0.586	1.00 (0.93–1.08)	0.958
Subsequent ARPI use	1.23 (0.59–2.57)	0.584	2.45 (0.75–7.99)	0.137
Subsequent cytostatica	1.02 (0.98–1.06)	0.408	1.05 (0.98–1.11)	0.143
Overall survival				
Radiotherapy	**0.41 (0.20–0.85)**	**0.016**	**0.14 (0.03–0.57)**	**0.006**
Age	1.05 (0.99–1.11)	0.087	1.02 (0.92–1.13)	0.697
Time interval initial diagnosis to PSMA-PET	0.99 (0.99–1.002)	0.118	0.99 (0.98–1.01)	0.837
Initial PSA	1.00 (0.99–1.01)	0.127	1.00 (0.99–1.01)	0.153
Gleason score	**2.05 (1.25–3.36)**	**0.005**	2.05 (0.76–5.52)	0.154
Initial tumor stage	1.01 (0.68–1.50)	0.961	0.36 (0.13–1.01)	0.052
Charlson comorbidity index	2.05 (0.96–4.41)	0.065	0.73 (0.18–3.01)	0.671
Duration of ADT until PSMA-PET	**0.99 (0.98–1.00)**	**0.040**	0.99 (0.97–1.003)	0.103
PSA at PSMA-PET	0.99 (0.93–1.06)	0.763	0.77 (0.58–1.03)	0.077
PSA doubling time	0.97 (0.89–1.04)	0.376	1.03 (0.91–1.15)	0.659
Subsequent ARPI use	0.56 (0.25–1.29)	0.176	0.46 (0.10–2.11)	0.318
Subsequent cytostatica	0.96 (0.82–1.14)	0.660	0.99 (0.83–1.20)	0.972

OS was significantly associated with Gleason score (HR = 2.05, 95% CI 1.25–3.36, *p* = 0.005), duration of ADT until PSMA-PET (HR = 0.99, CI 0.98–1.00, *p* = 0.040) and treatment group (HR for MDRT = 0.41, 95% CI 0.20–0.85, *p* = 0.016) in univariate testing. Upon multivariate testing, only radiotherapy remained significantly associated with OS (HR = 0.14, 95% CI 0.03–0.57, *p* = 0.006).

## Discussion

The present study suggests that patients with metachronous oligometastatic CRPC might benefit from PSMA-PET-guided MDRT to all metastatic lesions compared to standard treatment alone in terms of bPFS and OS.

Moreover, freedom from second line therapy could be prolonged for patients treated with additional MDRT compared to standard of care alone. This is a potentially interesting exploratory finding, that should however interpreted with care as subsequent treatment was very heterogeneous due to the retrospective nature of this study.

This positive outcome may be due to the potential of MDRT to eliminate clonally resistant metastases while ongoing ADT controls further disease progression, as hypothesized in previous studies^15,17^.

In terms of hormone sensitive prostate cancer patients there is evidence that local treatment has a positive impact on PFS and OS and can prolong the postponement of ADT^8,18^.n recent years, there has also been increasing research in the use of MDRT in castration-resistant PCa patients; encouraging results have been reported in terms of PFS, OS and freedom from second-line therapy^19,20^.

Table 4 summarizes studies on local therapy for ologometastatic/ oligoprogressive CRPC. There are two prospective trials with control groups: The randomized phase-II ARTO trial which included 157 patients and randomized patients to Arbiraterone alone or Arbiraterone plus additional SBRT. ARTO showed a significant improvement of progression free survival by SBRT. However, only around 25% of patients were staged with PSMA-PET. This is important as it has recently been shown that the chosen imaging modality strongly affects the oncological outcome in this setting with PSMA PET CT being superior to Choline PET and potentially also conventional staging^21^.

**Table 4:** Clinical studies on metastasis directed therapy in CRPC.

First author	Study type	Control group	PSMA-PET	Endpoint
Francolini^26^	Randomized prospective trial	Yes (Arbiraterone)	Approx.. 25%	N = 157 Biochemical response and progression free survival
Baron^42^	Retrospective multicenter	No	No information	N = 50 Median OS not reached, median PFS = 13 months
Chamois^43^	Retrospective multicenter	No	19.6%	N = 107 Median PFS 12.6 months
Pan^22^	Prospective singlecenter	Yes (patient decision)	100%	N = 29 (SBRT), n = 18 (control) Metastasis free survival significantly improved by SBRT HR 4.69
Zhang^23^	Prospective singlecenter	No	0%	N = 89 Median PFS 9.5 months
Lohaus^15^	Retrospective singlecenter	No	100%	N = 15 Median PFS 17.9 months
Henkenberens^20^	Retrospective singlecenter	No	100%	N = 42 Median PFS 12 months
Berghen^44^	Retrospective multicenter	No	47%	N = 30 Median PFS 10 months
Onal^24^	Retrospective multicenter	No	100%	N = 67 Median PFS 11 months

Therefore, the other prospective trial by Pan and colleagues is especially interesting^22^. In this trial all patients underwent FDG and PSMA PET for staging. Patients with oligoprogressive CRPC could choose either SBRT (n = 29) or continuation of ADT n = 18. The analysis revealed a strong improvement of metastasis free survival by additional local therapy. Other endpoints like OS have not been reported so far, probably due to relatively short follow-up of patients.

There is one third prospective single arm trial by Zhang and colleagues^23^. Their trial included 89 patients who received additional SBRT. The Median PFS was reported 9.5 months, however none of the patients was staged by PSMA-PET.

In addition to these prospective trial, there are several retrospective studies available on this topic as depicted in Table 4. These retrospective studies share the common limitation that a control arm is lacking. The studies published by Lohaus, Henkenberens and Onal have the advantage that all patients were staged with PSMA-PET^15,20,24^. These studies reported Median PFS between 11 and 18 months.

Looking at the results of our study, the median bPFS was quite favourable in both groups with 25 months after MDRT versus 23 months for patients receiving standard of care only.

Similar results were presented in a retrospective mono-institutional study by Mulderman et al. They reported a 16-month bPFS of 54% in patients, most with castration-resistant disease and ongoing ADT, treated with SABR to all metastatic sites^27^. However this study, is difficult to interpret as not all patients had CRPC.

In addition, one study has shown that in oligoprogressive CRPC patients, who already receive ARPI such as Abiraterone or Enzalutamide, MDRT may be able to prolong the time to further escalation of systemic therapy^25^. These studies are of retrospective nature with all its limitations. Although this important limitation holds also true for our study, to the best of our knowledge this is the first study that includes a control group that received standard of care treatment without MDRT.

In the our study we could identify radiotherapy as the only independent predictor for bPFS HR = 0.36 (*p* = 0.048) in multivariate analysis. It is quite notable that this is very similar to the ARTO trial, which reported a HR for progression of 0.35 (*p* < 0.001). In the ARTO trial, progression was defined as the time from the start of ARPI to biochemical or radiologic progression, initiation of subsequent treatment, or death^26^.

Regarding OS, the present study could show a significantly better outcome for patients treated with MDRT compared to standard of care alone. Although this finding should be interpreted carefully due to the small number of patients and retrospective nature, we still think that this is remarkable. This was feasible by analysing patients with PSMA-PET between 2014 and 2018, enabling a very long follow-up of all patients. Nonetheless, choosing patients with long follow-up is also a limitation as ARPI is now used at an earlier stage of disease.

Some other studies published data on OS, however with much shorter follow-up.In a multicenter retrospective study, oligometastatic CRPC patients treated with MDRT in addition to standard therapy showed a 3-year OS of 81.4%^29^.

In comparison, Triggiani et al. reported on 41 oligoprogressive CRPC patients who were treated with MDRT to all metastatic lesions detected by choline PET or CT plus bone scan, they showed a 2-year OS of 93.3%^30^.

In our study, a 2-year freedom from second-line therapy (FFSLT) rate of 73.5% (n = 25 of 34) was achieved in the MDRT arm compared to 28.6% (n = 6 of 21) in the control arm. This is an interesting finding as second line therapy regimes may be detrimental to quality of life of patients. Even more intriguing is the fact that patients received second line treatment at a later time while still maintaining favorable overall survival compared to the control group with earlier use of second line systemic treatment. This indicates that deferral of systemic treatment intensification by effective local treatment does not seem to endanger survival of patients.

 Henkenberens et al. reported a median second-line systemic treatment free survival (e.g. ARPI or docetaxel) of 15.0 months for oligometastatic CRPC patients (n = 42) treated with MDRT to metastatic sites, detected by PSMA-PET^20^.

Similarly, in a multicenter retrospective study, the median systemic therapy-free survival was 21.8 months. In this trial, 86 oligoprogressive CRPC patients were treated with MDRT to all metastatic lesions detected by Choline PET or CT plus bone scan. The systemic therapy-free survival (STFS) was defined as the time from the start of MDRT to the initiation of systemic therapy^16^.

One of the strengths of our study is that all patients receiving PSMA PET CT scans were meticulously screened and selected according to clearly specified parameters. In addition, all participants underwent PSMA PET for imaging modality. PSMA PET has proven to be an effective imaging technique in terms of detecting metastatic lesions at an early stage of prostate cancer and is superior to scintigraphy or CT based staging^31^. Another strength of this study is the presence of a control group and the relatively long duration of follow-up. This is especially important for PSMA PET staged patients, as this diagnostic method is relatively new and follow-up data often limited.

Nonetheless, there are also several important limitations of this study. Firstly, the retrospective design with the potential of selection bias between treatment arms. This is an important limitation, however based on comorbidities and tumor characteristics we have no indication that patients with standard of care treatment showed poorer performance compared to the MDRT group. The different pattern of metastases (more frequently bone metastases in MDRT group versus lymphonodal metastases in the control group) might indicate the different patient characteristics of the referring physicians. Patients with bone metastases are frequently treated with radiotherapy and this group seems therefore larger in the MDRT group, who was primary referred to PSMA PET CT by radiation oncologists. It is remarkable that the imbalance between M1a and M1b patients should be in favor of the control group, as M1a patients have lower rates of prostate cancer mortality compared to M1b patients^32^. Despite this, a favorable OS was observed in the MDRT group. A second limitation is the heterogeneous group of oligometastatic/oligoprogressive CRPC patients with different patient and treatment characteristics. This is probably a general limitation in this specific setting. A more important limitation is the inclusion of patients with untreated primary tumors. This is an important limitation, however it has to be noted that this was only a minority of patients (10 out of 55 patients) and all of these patients presented additional metastases.

Thirdly, the lack of predefined study protocol with different radiation dosage schemes and methods is a minor limitation as all patients received relatively high doses of radiotherapy.

Another limitation regards the standard of care treatment of patients. At the moment ARPI is increasingly used in earlier stages of disease. Only one patient in our study had received ARPI prior to PSMA-PET. An important and open question is certainly the timing of treatment escalation (ARPI and additional local treatment), that cannot be adressed by our current study.

There is a need to further identify significant predictors in mCRPC patients to better understand which patients will benefit most from additional MDRT.

The results of several ongoing prospective trials addressing our research question are eagerly awaited^33–35^.

MDRT is a well-tolerated treatment option with low toxicity in oligometastatic CRPC patients. Our comparison with patients receiving standard of care suggests an improved bPFS, OS and freedom from second-line therapy.

The favorable toxicity and efficacy indicates that MDRT can be considered in the decisionmaking when treating oligometastatic CRPC patients.

Predictive factors also have to be evaluated to select patients who are likely to benefit from MDRT to all metastatic lesions.

Further studies in oligometastatic CRPC patients are needed to confirm our results.

## Methods

### Patients and imaging

**Fig. 3:**
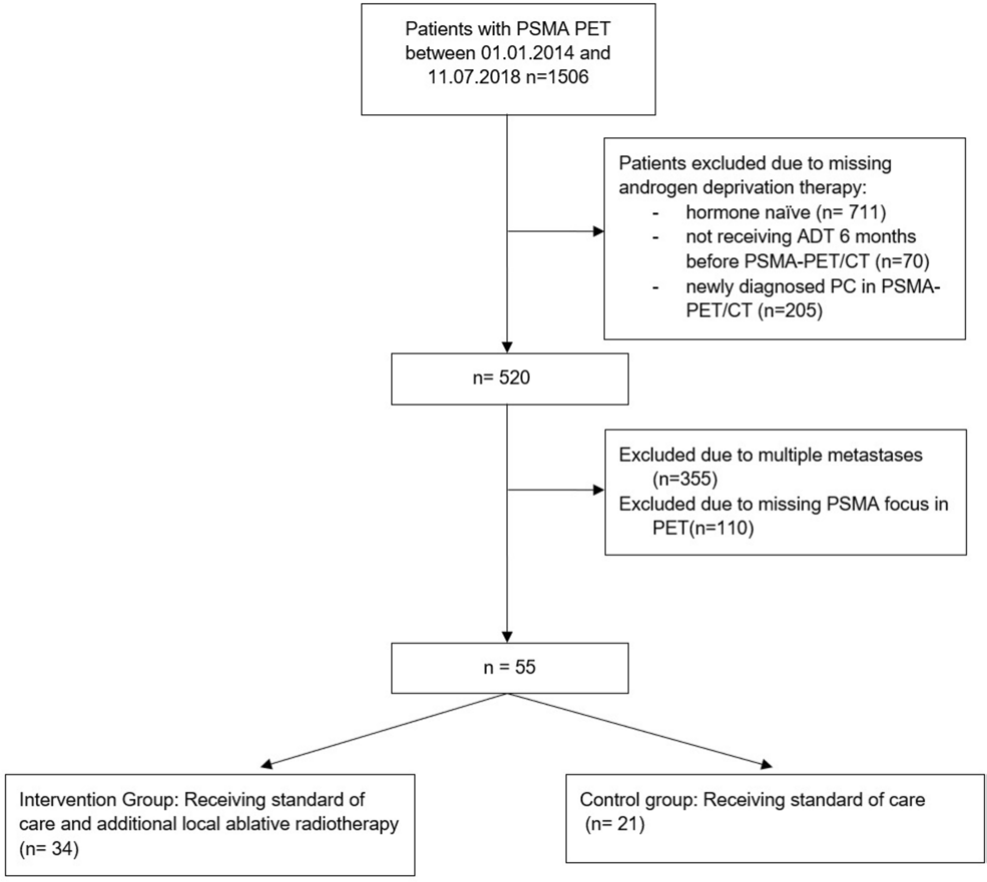
Flowchart showing the workup of the presented analyses of all patients within our institutional database.

In this retrospective monoinstitutional study we analysed all PSMA-PET diagnostic reports written by nuclear Medicine physicians/ radiologists between January 2014 and July 2018 using the database of our institution, see flowchart Fig. 3. Eligible patients had an oligometastatic/oligoprogressive state of their disease, defined as 1–5 metastases detected by PSMA-PET according to recommendations of the European Socitey for Radiotherapy and Oncology (ESTRO) and European Organisation for Research and Treatment of Cancer (EORTC) consensus^36^. Since PSMA-PET was not routinely used before initiation of ADT, there was only conventional initial imaging available. Patients with more than 5 lesions in initial imaging were excluded in this analysis to avoid heterogeneity of the population. Moreover, all participants had received ADT at least 6 months before PSMA-PET and their prostate cancer had progressed to castration resistant. These patients were in an metachronous oligoprogressive state of their disease as their biochemical relapse emerged during ADT^37^.

Patients were excluded due to missing androgen deprivation therapy or not receiving ADT six months before PSMA-PET. Further exclusion criteria were multiple metastases or missing PSMA focus in PSMA-PET. UICC classification was used to graduate prostate cancer patients^11^. PSA Doubling Time Calculator MDApp was applied calculating PSA doubling time for all patients^38^. The Charlson Comorbidity Index (ICC) served to rate comorbidities^39^.

### Treatment

All patients underwent PSMA-PET. The therapy after baseline PSMA-PET was individually chosen by the treating physicians. According to the therapeutic approach the patients were divided into two arms. Patients receiving MDRT to all PSMA-positive metastases and standard of care (MDRT group). The control group was defined as patients who were treated with standard of care only (control group). Standard of care always included continuation of ADT and additional treatment escalation with ARPI, chemotherapy or other agents, according to individual preferences of patients and treating physicians. MDRT was most frequently performed by SBRT (stereotactic body radiation therapy) or high dose fractionated EBRT (external beam radiation therapy) or a combination of both within 3 months after baseline PSMA-PET. SBRT respectively EBRT were delivered in different schedules according to clinical practice. The prescription of radiotherapy was according to physicians´ preference and tumor characteristics. High dose EBRT was defined as doses above 45 Gray 2 Gray equivalent dose with alpha/beta ratio of 3. Details on applied radiotherapy schedules can be found in supplementary table 1. For toxicity grading Common Terminology Criteria for Adverse Event version 4.0 was used.

### Statistical analysis and follow up

For comparison of both groups Kaplan Meier-survival curves were applied to calculate estimated biochemical progression free survival (bPFS), overall survival (OS), freedom from second line therapy (FFSLT), freedom from ARPI therapy (FFARPI) and freedom from chemotherapy (FFC). bPFS was calculated as recommended by the Prostate Cancer Clinical Trials Working Group as an PSA increase of 2 ng/ml compared to PSA Nadir^40^. Patients with initial ARPI or cytostatic therapy were excluded in the analyses of the respective endpoint. Univariate and multivariate cox regression was used to evaluate prognostic parameters considering bPFS and OS. Kaplan–Meier estimates were plotted for survival estimates of different groups. Statistical analyses were created by SPSS software, version 27 (IBM Corporation, Armonk, NY, USA).

Follow up data was provided by a hospital-based data-acquisition system, patient files, treating physicians and patient contact. Biochemical progression was defined as PSA level at baseline PSMA-PET + 2 ng/ml according to the recommendations of the Prostate Cancer Clinical Trails Working Group (PCWG3)^41^. The study was approved by the local Ethics Commission Charité Nr EA4/168/16. All patients had given their written informed consent for data use, all methods were performed in accordance with the Declaration of Helsinki.

## Supplementary Information

Supplementary Information.

## Data Availability

The datasets generated during and/or analyzed during the current study are available from the corresponding author on reasonable request.
