# Supplementary material for: Metastasis directed radiotherapy versus standard of care for PSMA-PET diagnosed oligometastatic/oligoprogressive castration resistant prostate cancer
Source: Sci Rep. 2025 Jul 25;15:27153. doi: 10.1038/s41598-025-09622-7 (PMC12297292; doi:10.1038/s41598-025-09622-7)
Supplement: Supplementary file 1 — Supplementary Information. [file 41598_2025_9622_MOESM1_ESM.docx]

**Supplementary table 1:** Details on applied radiation therapy. Patients that were treated with external beam radiotherapy (EBRT) received helical tomotherapy (Accuray, USA) either normofractionated or moderately hypofractionated. patients that were treated with stereotatctic body radiotherapy were treated at the Novalis™ radiosurgery system (Varian, USA; Brainlab, Heimstetten, Germany) or by Cyberknife (Accuray, USA).

| **Radiotherapy schedules** | |
| --- | --- |
| EBRT (Tomo) | n= 13 (38,24%) |
| SBRT (Novalis/ Cyberknife) | n= 16 (47,06%) |
| EBRT and SBRT | n= 5 (14,71%) |
| Number of irradiated lesions | n= 81 |
| **EBRT fractionation schemes:** | |
| Conventional fractionated (1.7-2.2 Gy) | n=13 (38.24 %) |
| Hypofractionated (3.0 or 3.5 Gy) | n=5 (14.70 %) |
| **SBRT fractionation schemes:** | |
| Stereotactic fractionated (most commonly 3 times 10 Gray or 13 times 3.8 Gray) | n= 14 (41.18%) |
| Single fraction | n= 8 (23.53%) |
